# A review of patch-test data over the period of eight years in the allergy and immunology department in Qatar

**DOI:** 10.5339/qmj.2023.sqac.4

**Published:** 2023-11-19

**Authors:** Sally Khalil, Sherin Rahim, Hassan Mobayed, Maryam Al-Nesf

**Affiliations:** ^1^Allergy and Immunology Division, Department of Medicine, Hamad Medical Corporation, Doha, Qatar Email: SKhalil3@hamad.qa

## Abstract

Background: Patch testing is the primary diagnostic approach for contact dermatitis,^1^ an inflammatory skin reaction caused by exposure to external irritants. The pathophysiology of contact dermatitis may entail an immunological response (hypersensitivity type IV), a non-immunological response (irritant contact dermatitis), or a mix of the two. The diagnosis of contact dermatitis requires a correlation between a positive patch test and clinical relevance.^2^ This study aims to determine the prevalence of allergy sensitization among adults in Qatar and the allergens most frequently associated with positive patch test findings.

Methods: Retrospective analysis used patch testing data from 2015 to 2022.

Results: Of the 87 patients tested, 43 had at least one positive reaction (mean age 41.7; range 19–68). Females were 33 of the total patients (76.7%). Thirteen (30%) patients had two or more positive reactions. The most common allergen groups associated with positive patch test reactions were nickel sulfate no. 12 (27.9%), and all reactants were female. followed by gold sodium thiosulfate no. 10 (23.3%, F:M = 2.3), p-phenylenediamine no. 10 (23.3%, F:M = 1.5), and p-tert-butylphenol formaldehyde resin no. 7 (16.2%, F:M = 6). Twenty-six reactants had one or more allergic disorders (allergic rhinitis, asthma, drug allergy, insect bite allergy, or chronic idiopathic urticaria), and 11 had atopic dermatitis.

Conclusion: Triggering agents for contact dermatitis vary among geographic regions and populations. This study gives an idea of the allergens that are the most common sensitizers among the contact dermatitis population in the adult allergy clinic in Qatar.

## Figures and Tables

**Table 1.** Substances tested in the Adult Allergy Clinic in Qatar. T.R.U.E. TEST Allergen includes 35 common allergens and negative control.

**Panel 1.3**	**Panel 2.3**	**Panel 3.3**
Nickel Sulfate	p-tert-Butylphenol Formaldehyde Resin	Diazolidinyl Urea
Wool Alcohols	Epoxy Resin	Quinoline Mix
Neomycin Sulfate	Carba Mix	Tixocortor-21-Pivalate
Potassium Dichromate	Black Rubber Mix	Gold Sodium Thiosulfate
Caine Mix	Cl+Me- Isothiazounone (MCl/MI)	Imidazolidinyl Urea
Fragrance Mix	Quatemium-15	Budesonde
Colophony	Methyldibromo Glutaronitrile	Hydrocortzone-17-Butyrate
Paraben Mix	p-Phenylenediamine	Mercaptobenzothiazole
Negative Control	Formaldehyde	Bacitracin
Balsam of Peru	Mercapto Mix	Parthenolide
Ethylenediamine Dihydrochloride	Thimerosal	Disperse Blue 106
Cobalt-Dichloride	Thiuram Mix	Bronopol
